# Universal Gestational Age Effects on Cognitive and Basic Mathematic Processing: 2 Cohorts in 2 Countries

**DOI:** 10.1016/j.jpeds.2015.02.065

**Published:** 2015-06

**Authors:** Dieter Wolke, Vicky Yu-Chun Strauss, Samantha Johnson, Camilla Gilmore, Neil Marlow, Julia Jaekel

**Affiliations:** 1 Department of Psychology, University of Warwick, Coventry, United Kingdom; 2 Warwick Medical School, University of Warwick, Coventry, United Kingdom; 3 Center for Statistics in Medicine, University of Oxford, Oxford, United Kingdom; 4 Department of Health Sciences, University of Leicester, Leicester, United Kingdom; 5 Mathematics Education Center, Loughborough University, Loughborough, United Kingdom; 6 University College London, London, United Kingdom; 7 Department of Developmental Psychology, Ruhr-University Bochum, Bochum, Germany

**Keywords:** BLS, Bavarian Longitudinal Study, EP, Extremely preterm, GA, Gestational age, K-ABC, Kaufman-Assessment Battery for Children, MPC, Mental processing composite, RMSE, Root mean square error, SES, Socioeconomic status, SGA, Small for GA, UK, United Kingdom

## Abstract

**Objective:**

To determine whether general cognitive ability, basic mathematic processing, and mathematic attainment are universally affected by gestation at birth, as well as whether mathematic attainment is more strongly associated with cohort-specific factors such as schooling than basic cognitive and mathematical abilities.

**Study design:**

The Bavarian Longitudinal Study (BLS, 1289 children, 27-41 weeks gestational age [GA]) was used to estimate effects of GA on IQ, basic mathematic processing, and mathematic attainment. These estimations were used to predict IQ, mathematic processing, and mathematic attainment in the EPICure Study (171 children <26 weeks GA).

**Results:**

For children born <34 weeks GA, each lower week decreased IQ and mathematic attainment scores by 2.34 (95% CI: −2.99, −1.70) and 2.76 (95% CI: −3.40, −2.11) points, respectively. There were no differences among children born 34-41 weeks GA. Similarly, for children born <36 weeks GA, mathematic processing scores decreased by 1.77 (95% CI: −2.20, −1.34) points with each lower GA week. The prediction function generated using BLS data accurately predicted the effect of GA on IQ and mathematic processing among EPICure children. However, these children had better attainment than predicted by BLS.

**Conclusions:**

Prematurity has adverse effects on basic mathematic processing following birth at all gestations <36 weeks and on IQ and mathematic attainment <34 weeks GA. The ability to predict IQ and mathematic processing scores from one cohort to another among children cared for in different eras and countries suggests that universal neurodevelopmental factors may explain the effects of gestation at birth. In contrast, mathematic attainment may be improved by schooling.

See related article, p 1417

Around 15 million babies worldwide (∼10% of all births) are born preterm (<37 weeks gestational age [GA]) each year. Changes in reproduction patterns and improved neonatal medicine have led to increased numbers of moderately (32-33 weeks GA) and late preterm (34-36 weeks GA) births and increased survival rates of those born very preterm (<32 weeks GA). Despite improved neonatal care, prematurity remains the leading cause of infant mortality and long-term morbidity today,^1^ and the high prevalence of cognitive problems (>20%) in preterm populations has not changed over the last 2 decades.^2^

Studies suggest that delivery at any gestation other than full-term may confer an insult to brain development^3^ rendering survivors at risk for adverse cognitive and educational outcomes, particularly in mathematics.^4-6^ It remains controversial whether the dose response effect of GA on early mathematical abilities is linear^6^ or curvilinear.^7^ Emerging evidence from different cohorts demonstrate a significant impact of GA at birth on basic cognitive abilities (eg, IQ, mathematic processing)^8,9^ and mathematic attainment,^4,6,10^ but there is uncertainty about its specific nature and magnitude. The relationship of GA with cognitive and educational outcomes may be affected by differences in neonatal care across cohorts or eras of care, particularly across the 1980s and 1990s, with increased survival following advances in surfactant treatment, ventilation techniques, or nutrition.^1,2,11,12^ Furthermore, cognitive abilities and attainment may be affected by socioeconomic status (SES) and early education.^13,14^

We investigated the association of GA with cognitive ability (IQ), basic mathematic processing, and mathematic attainment assessed during second grade of elementary school (8 years of age) in the Bavarian Longitudinal Study (BLS) cohort born 1985/1986 in the South of Germany at 27-41 weeks GA. We then used the regression functions identified in the BLS sample to predict IQ, basic mathematic processing, and mathematic attainment assessed at second grade in the United Kingdom (UK) (6 years) and 11 years of age using the same tests in the EPICure national cohort of extremely preterm (EP) children born in 1995 in the whole of the UK and Ireland at 23-25 weeks GA.

We, first, hypothesized that the effects of GA on IQ and basic mathematic processing^8,15^ are universal; that is, similar deficits would be found across cohorts assessed in different countries and during different eras of neonatal care.^2^ Second, we hypothesized that mathematic attainment^9,16^ may be susceptible to country specific schooling and that outcomes may, thus, differ between cohorts; that is, prediction from one cohort to another may be less accurate compared with predictions of basic cognitive abilities.

## Methods

Two prospective geographically defined birth cohorts were included, the BLS and the EPICure study. Descriptive characteristics of the BLS and EPICure study participants are in Table I.

### BLS Cohort

The enrollment procedures have been described in detail elsewhere.^17-19^ A total of 7505 infants (10.6% of all live births) who were born between January 1985 and March 1986 in Southern Bavaria, Germany, and required admission to a children's hospital within the first 10 days of life were invited to participate in this study (index children). In addition, 916 term-born infants who received normal postnatal care were identified in the same hospitals. Ethical approval was obtained from the Ethics Committee of the University of Munich Children's Hospital and the Bavarian Health Council (Landesärztekammer). Analyses for this study use follow-up data at 8 years. At this age, we assessed 336 very preterm survivors and a sample of 1169 children born >31 weeks GA stratified by child sex, family SES, and degree of neonatal risk. Of these, 156 children could not complete the full battery of tests and were excluded. Data from 20 EP children (<27 weeks GA) were excluded as the number was too small to allow for appropriate statistical estimates. Finally, 40 children born post-term (>41 weeks GA) were excluded given the established association with adverse developmental outcomes.^20^ The final BLS sample for this study thus comprised 1289 children born between 27 and 41 weeks GA. All tests were standardized according to 584 children born full term (39-41 weeks) within the sample (298 receiving normal postnatal care and 286 index full-term children) who were followed to 8 years.

### EPICure

The EPICure study included EP infants who were born before 26+0 weeks GA in the UK and Ireland from March through December 1995. The sampling of the study population has been described previously.^10,21^ Ethics approval was granted by the Trent Multicenter Research Ethics Committee. In total, 241 and 219 survivors were followed to age 6 and 11 years, respectively. Children with severe physical disability who could not complete the tests were excluded (n = 48), leaving 171 EP children. Cognitive abilities and mathematics attainment were assessed at 6 years and mathematic processing at 11 years. All tests were standardized according to full-term control children (37-41 weeks gestation) from the same classes in mainstream schools at 6 (n = 160) and 11 years of age (n = 153).^22,23^

### Measures

In both studies, GA (completed weeks) was calculated from maternal reports of the last menstrual period and serial ultrasounds during pregnancy.^23,24^ In both studies, psychologists assessed cognitive abilities using the Kaufman-Assessment Battery for Children (K-ABC).^25,26^ This yielded a mental processing composite (MPC) score indicating general cognitive ability (IQ).

**Figure 1:**
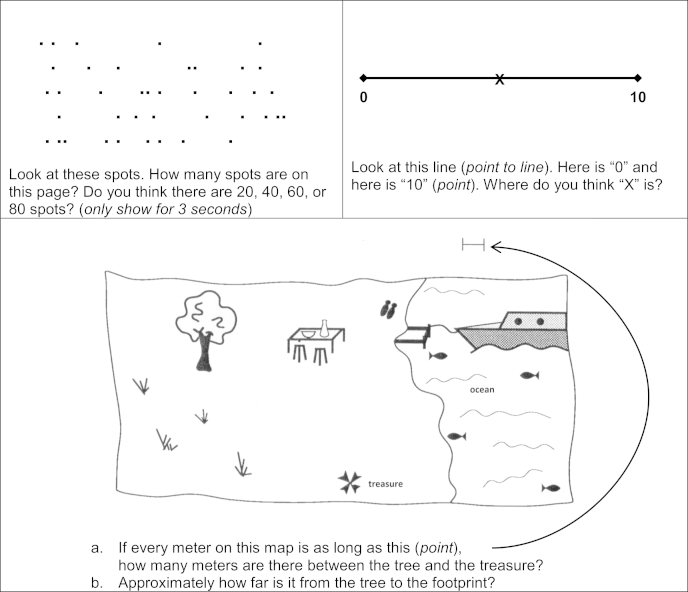
Mathematics Estimation Test example items.

Children in both studies were administered a Mathematics Estimation Test^9,27^ at age 8 (BLS) and 11 (EPICure) years, respectively. Tasks were presented to children in book form with 12 items assessing the estimation of dot array and number line magnitude, as well as judgments of approximate length and distance (Figure 1; available at www.jpeds.com). Item responses were scored for accuracy and summarized into a total score. Test scores were standardized based on term controls in each study separately (standardized control mean 100; SD 15).

In both studies, the age-appropriate K-ABC arithmetic subtest (separate from the MPC) assessed children's attainment in mathematics.^25,26^ At the time of the K-ABC assessment, children in both cohorts had received, on average, 2 years of formal school education. For the purpose of comparison between the 2 cohorts, MPC and K-ABC mathematics scores were standardized according to the full-term control children in each study separately (standardized control mean 100; SD 15). Children who could not be assessed because of severe cognitive disability were assigned a score of 39 for IQ and mathematics attainment (ie, 1 point below the minimal possible assessment score).

Analyses were controlled for family SES, child sex, and small for GA (SGA) birth. Infants were classified as SGA if they weighed less than the sex specific 10th percentile for their GA according to the national German standard weight charts (1985-1986)^28^ and based on the UK child growth foundation charts in EPICure.^29^ Family SES was classified into 3 categories corresponding to high, medium, and low using parental education and occupation.^30,31^

### Statistical Analyses

Missing data was imputed with full information maximum likelihood estimates.

#### BLS

In order to identify the best fitting model for GA effects on outcomes in the BLS cohort, piecewise linear regressions were fitted for IQ, mathematic processing, and mathematic attainment using the STATA v 12 nonlinear functions (Stata Corp, College Station, Texas) and Taylor change-point analysis tools. This method was selected based on previous findings of a nonlinear effect of GA on cognitive and mathematic outcomes.^7,32^ Piecewise regressions were used to identify the week of GA at which test performance differed significantly above and below a change point. Final models were adjusted for family SES, child sex, and SGA.

#### EPICure

Accuracy of predicted IQ, mathematic processing, and mathematic attainment scores for EP children was evaluated by inserting their observed scores into the piecewise regressions fitted to the BLS sample. The 50% and 75% prediction intervals (ie, predicted scores ± 0.67 (Z0.52) and 1.04 (Z0.252), root mean square errors [RMSEs]) were then calculated. RMSEs indicate the SDs of the residuals (ie, the difference between observed and predicted scores). The precision of these predictions was examined by the range of EPICure observed scores (ie, 25th-75th percentiles) that fell within these 50% (1 RMSE) and 75% (2 RMSEs) prediction intervals.^33^ A prediction was assumed to be very precise if the 25th-75th percentiles of observed scores were covered within the 50% prediction interval. All models were controlled for family SES, child sex, and SGA birth.

## Results

### The Effect of Birth at 27-41 Weeks GA on IQ, Mathematic Processing, and Mathematic Attainment in the BLS Cohort

**Figure 2:**
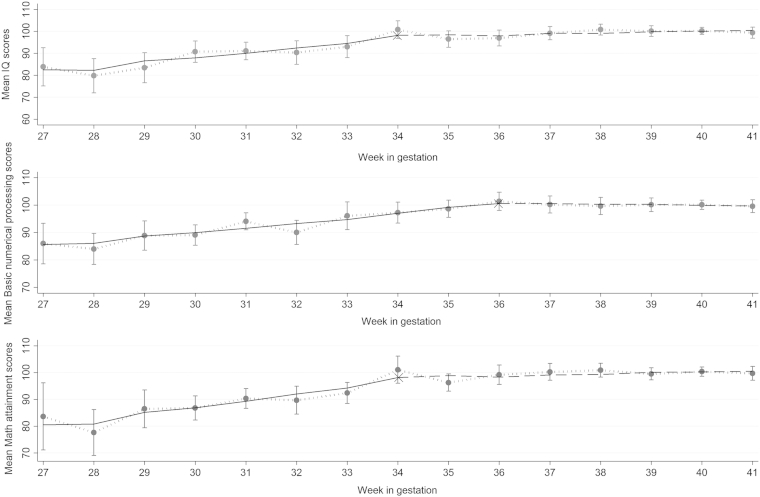
Observed and predicted mean change in outcomes according to GA at birth in the BLS (Germany; 27-41 weeks GA). *Grey vertical lines*: 95% CIs of observed means (*circles*); *X*: GA change points; *black solid horizontal lines*: predicted means below the GA change point; *dashed horizontal lines*: predicted means above the GA change point.

Piecewise regressions showed that GA exerted differential effects on IQ and mathematics attainment below vs above 34 weeks (95% CI: 31 weeks, 37 weeks; and 32 weeks, 36 weeks, respectively) and on basic mathematic processing below vs above 36 weeks (95% CI: 34 weeks, 38 weeks). Table II and Figure 2 show that after controlling for SES, sex, and SGA, children's IQ and mathematics attainment scores decreased by 2.34 points (95% CI: −2.99, −1.70) and 2.76 points (95% CI: −3.40, −2.11) with each lower week of GA below 34 weeks, respectively. There were no significant differences among children born at 34-41 weeks GA for both outcomes. Basic mathematic processing scores decreased by 1.77 points (95% CI: −2.20, −1.34) with each week of GA below 36 weeks, and there was no significant effect of GA for children born at 36-41 weeks. In addition to GA, low SES had strong negative effects on outcomes. For example, the effect of low SES on IQ was equivalent to that of 5 weeks of GA below the change point (34 weeks). On average, SGA birth had negative effects on all outcomes across the gestation spectrum and girls had worse mathematics attainment and basic mathematic processing scores than boys (Table II).

### Predicted Performance of EPICure Children According to BLS Regression Functions

**Figure 3:**
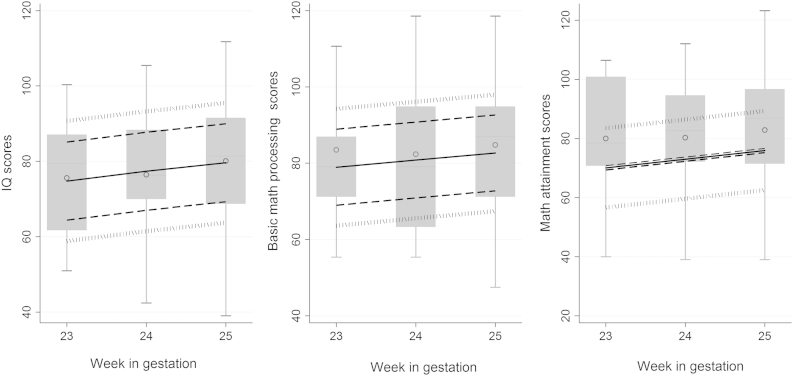
EPICure Study observed score distributions (*box plots*^∗^) with predicted mean scores (*solid lines*) and 50% (*dashed lines*) and 75% (*dotted lines*) prediction intervals based on the BLS cohort. Precision of prediction was examined by calculating the percentiles of box plots within prediction intervals and show that observed IQ and basic mathematic processing scores were mostly covered within the 50% prediction interval. ^∗^The bottom and top of each box are the 25th and 75th percentiles of observed scores, respectively. The line in the middle is the 50th percentile and *hollow circles* are observed mean scores.

**Figure 4:**
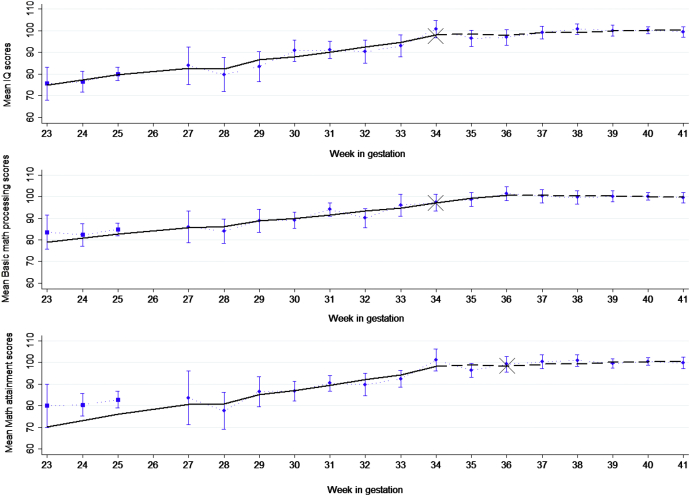
Observed and predicted mean change of outcomes according to GA at birth in the EPICure Study (23-25 weeks GA) and BLS (27-41 weeks GA). *Blue vertical lines*: 95% CIs of observed means (*circles*: BLS and *squares*: EPICure); *X*: GA change points; *black solid horizontal lines*: predicted means below the GA change point; *dashed horizontal lines*: predicted means above the GA change point.

Accuracy of predicted IQ, mathematic processing, and mathematic attainment scores for EP children was evaluated by inserting their observed scores into the piecewise regressions fitted to the BLS sample. Figure 3 shows distributions of EPICure Study children's observed scores (box plots) vs their predicted scores (lines) with 50% and 75% prediction intervals based on the BLS data. Both observed IQ and basic mathematic processing scores between 25th and 75th percentiles were mostly covered within the 50% prediction interval (Figures 3 and 4; Figure 4 available at www.jpeds.com), showing observed and predicted scores by GA in both BLS and EPICure children. Thus, consistent with hypothesis 1, BLS children's scores (27-41 weeks GA) allowed accurate prediction of IQ and basic mathematic processing scores of children born at 23-25 weeks GA in another country one decade later.

In contrast, the top one-half of the observed EPICure mathematics attainment scores were only within the range of the 75% prediction interval; they deviated more than 1 RMSE from the predicted scores. Thus, EPICure children had higher mathematics attainment scores than was predicted from BLS data.

## Discussion

This study investigated the effect of birth across the whole gestation spectrum on cognitive abilities and mathematics attainment in middle childhood. The relationships between GA and outcomes were best fitted using piecewise regressions. These indicated deficits in IQ and mathematics attainment for children born <34 weeks GA and in basic mathematic processing abilities for children born <36 weeks GA. In addition, regression functions that were identified in the BLS sample accurately predicted EP children's IQ and basic mathematic processing scores in the EPICure study; however EP children's mathematic attainment in the EPICure study was better than predicted by performance of the BLS cohort.

First, these results provide evidence for a universal effect of GA at birth on long-term cognitive and basic mathematic processing abilities. Using data obtained from children born at 27-41 weeks gestation in Germany in 1985/1986, we successfully predicted IQ and mathematic processing scores in EP children born at 23-25 weeks gestation in the UK and Ireland in 1995. Given the particular decade that elapsed between recruitment of the 2 cohorts, EPICure children received pioneering new treatments such as surfactant administration that highly increased survival of EP children. Despite this, there was no equivalent improvement in their basic cognitive abilities. This is consistent with recent findings that compared 2 EP cohorts born in 1995 and 2006 that showed increased survival of EP infants but no improvement in neurodevelopmental outcomes.^2^ The precision of these predictions across different populations, decades, and health care systems indicates that underlying neurodevelopmental,^32^ rather than childhood environmental factors, may explain adverse effects of preterm birth on basic cognitive abilities. Accordingly, recent neuro-imaging studies have indicated changes in brain structure, function, and connectivity in relation to gestation at birth.^34-36^ Future studies that include neuro-imaging across cohorts may provide more direct evidence of similarly altered brain development. Our findings suggest that despite significant improvements in neonatal intensive care, there is considerable temporal and cross-national consistency in long-term cognitive abilities, at least in high income countries such as Germany and the UK. Increased survival, thus, provides no indication of improved cognitive function among children born moderately or very preterm.

Second, EPICure study children had higher mathematics attainment scores than were predicted by BLS data. Mathematics attainment was measured when children in both cohorts had received, on average, 2 years of formal schooling. There are, however, some important differences between the study populations' educational contexts. In the UK, children must enter compulsory schooling by 5 years of age, which is usually preceded by a year in reception class. Moreover, most children with special educational needs are admitted to mainstream school and receive extra and often individual help within class. Given the UKs inclusive education policies, only children with severe disabilities are admitted to special schools. Within the EPICure Study, only 6 (3.5%) EP children in the dataset used for analysis were in special schools. In contrast, in Germany, children had formal school entry assessments by community pediatricians that were used to stream children before entering elementary school. Those who passed the school entry tests entered elementary school in September after their 6th birthdays. Those who failed the school entry examination were either delayed for one year, (ie, entered school a year later at age 7 years) (96 children [7.4%] of the whole BLS sample [27-41 weeks GA]; 77 out of 319 children born <34 weeks GA [24.1%]) or were directly streamed into special schooling (73 children [5.7%] of the whole BLS sample; 44 out of 319 children born <34 weeks GA [13.8%], respectively). Thus German preterm children who often have mathematics achievement problems^9^ are less likely to receive support at mainstream school level. These discrepancies between the UK's and Germany's education systems may explain why EPICure children were doing much better in mathematics attainment than was expected.

**Figure 5:**
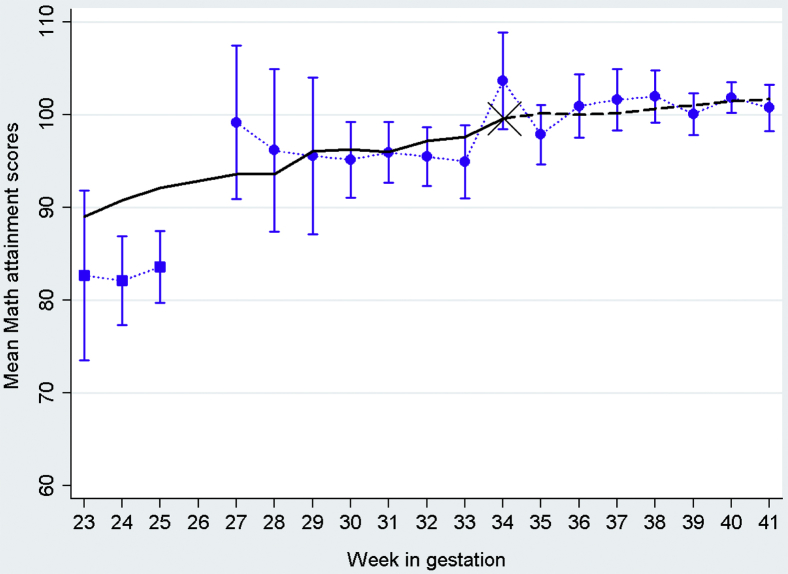
Observed and predicted mean change of mathematic attainment scores only for children in mainstream schools according to GA at birth in the BLS (N = 1094; 27-41 weeks GA) and in the EPICure Study (N = 165; 23-25 weeks GA). *Blue vertical lines*: 95% CIs of observed means (*circles*: BLS and *squares*: EPICure); *X*: GA change points; *black solid horizontal lines*: predicted means below the GA change point; *dashed horizontal lines*: predicted means above the GA change point.

In order to evaluate to what extent streaming into special schooling and delayed school entry may have accounted for German children's mathematic underachievement, we repeated our analyses only including BLS children who had entered mainstream school at the age appropriate time; EPICure children's observed mathematic attainment scores were now much lower than predicted (Figure 5; available at www.jpeds.com). This strongly suggests that special help within mainstream school may help children to attain mathematics abilities beyond their general cognitive abilities.^37^ The late preschool and early school years may represent a sensitive time for acquiring mathematical skills and preterm children may be highly sensitive to teacher or parent interventions at this time.^8,38^

Over and above the significant effect of GA, SES strongly predicted children's cognitive and mathematics abilities. The effect of growing up in a low SES family was equivalent to that of 2 (for basic mathematic processing) to 5 (for IQ) weeks decrease in GA for children born with a GA below the respective change points. Thus, our study confirms previously shown powerful influences of the social environment on preterm and full-term individual's long-term cognitive and educational outcomes.^39^ Further analyses indicated that there was no interaction effect between SES and GA, but rather an additive detrimental effect of low SES on preterm children's cognitive and educational outcomes, as has been shown before.^17^ Thus, low SES has similar adverse effects on cognitive and mathematics abilities for children born across the whole gestation spectrum.

This study evaluated the long-term effects of preterm birth across the whole gestation spectrum and cross validated findings in another cohort while controlling for key confounders. In both studies, children's abilities were assessed with the same standardized tests allowing for direct comparison across cohorts. To control for country specific impacts or the Flynn effect^40^ of increasing standardized test scores over time, the performance of preterm children was standardized according to term-born children recruited at birth in the BLS and classroom controls in the EPICure study.^18^ To estimate the association of gestation with outcome measures, those who were admitted to a children's hospital (index children) or had normal postnatal care between 39 and 41 weeks were combined in the BLS. These 2 full-term groups did not differ in basic mathematic processing and mathematics attainment scores but slightly in IQ (99.1 and 101.6; mean difference: −2.5 [95% CI: −4.6, −0.4]). Thus, overall IQ may have been slightly underestimated in the full-term range in the analysis but weighting did not alter prediction results. In both samples, K-ABC assessments were administered when children were at the same point in their school careers (ie, age 8 years in the BLS and age 6 years in EPICure, thus, they all had 2 years of school experience). The Mathematics Estimation Test was, however, administered when BLS children were 8 years and EPICure children were 11 years old. Although this timing difference may be seen as a caveat, the ability to nevertheless predict EPICure children's scores on the basis of an assessment done in another country at a different age and time point in children's school careers further strengthens the validity of our findings. Although our results suggest a universal effect of GA on childhood outcomes our findings are solely based on 2 European studies in high income countries which, despite some differences, may also present a number of similarities. In order to confirm the universal effect described here future studies should cross-validate our findings using non-European preterm samples. With regard to SGA classifications, the BLS and EPICure cohorts both used nationally appropriate growth chart samples. However, the generally small number of EP children born SGA in the EPICure study, a study of children at the limits of survival at the time, may be due to growth approximations in the charts at the time rather than actual growth chart data. Finally, IQ and math processing and attainment were assessed between 6 and 11 years. Future studies may include longer follow-up. However, both IQ and math tests have been shown to be highly predictive of outcomes in adulthood and even old age.^41,42^

The ability to predict long-term outcomes in general cognitive abilities and basic mathematic processing from one national cohort to another, both over time and with different neonatal services and social and education systems, suggests that neurodevelopmental rather than childhood environmental factors explain the long-term effects of gestation at birth. The finding that EPICure children had higher mathematic attainment scores than predicted suggests that national differences in elementary education may have substantial effects on preterm children's educational attainment chances despite similar general cognitive functioning. This may warrant further research including randomized controlled trials of tailored education interventions for preterm children.

## Figures and Tables

**Table I:** Descriptive characteristics of BLS and EPICure children included in analyses

	BLS children (N = 1289)	EPICure children (N = 171)
IQ	96.97 (16.70)	78.63 (16.62)
Basic mathematic processing	97.63 (15.58)	83.99 (16.12)
Mathematic attainment	96.87 (16.82)	81.84 (19.84)
GA	36.52 (3.94)	24.53 (0.66)
Sex (boys)	655 (50.81%)	74 (43.27%)
Age	8.34 (0.23)	6.28 (0.46)
SES		
High	386 (29.95%)	67 (45.58%)
Medium	485 (37.63%)	34 (23.13%)
Low	418 (32.43%)	46 (31.29%)
SGA	325 (25.21%)	14 (8.19%)

Data are presented as mean (SD) for numerical variables or numbers (percentages [%]) for categorical variables. Please note that EPICure children's basic mathematic processing abilities (Mathematics Estimation Test) were assessed at 11 years of age (mean = 10.91 [SD = 0.37]).

**Table II:** The association of GA with cognitive and mathematic performance in the BLS cohort (N = 1289) adjusted for child sex, family SES, and SGA status

	Regression coefficient *β* (95% CI)		
	IQ	Basic mathematic processing	Mathematic attainment
GA change point	34	36	34
<GA change point∗	−2.34 (−2.99, −1.70)	−1.77 (−2.20, −1.34)	−2.76 (−3.40, −2.11)
>GA change point∗	0.16 (−1.45, 1.78)	−0.33 (−1.68, 1.01)	0.16 (−1.46, 1.78)
Females	−1.24 (−2.89, 0.42)	−2.83 (−4.45, −1.21)	−5.01 (−6.67, −3.36)
High SES	1	1	1
Medium SES	−6.57 (−8.61, −4.54)	−2.97 (−4.96, −0.98)	−6.41 (−8.45, −4.37)
Low SES	−11.85 (−13.95, −9.75)	−4.96 (−7.02, −2.90)	−9.28 (−11.39, −7.17)
SGA	−5.48 (−7.44, −3.53)	−2.79 (−4.71, −0.88)	−5.29 (−7.24, −3.32)

∗ Coefficient *β* for each GA relative to GA change point.
